# Naringin in cholestatic liver ischemia-reperfusion injury

**DOI:** 10.1590/acb404225

**Published:** 2025-06-06

**Authors:** Arif Aslaner, Kemal Eyvaz, Erhan Aydemir, Şenay Yıldırım, Kübra Kılıç Kartal, Hamit Yaşar Ellidağ, Uğur Doğan

**Affiliations:** 1 University of Health Sciences – Antalya Training and Research Hospital – Department of Organ Transplantation – Antalya, Turkey.; 2 University of Health Sciences – Antalya Training and Research Hospital – Department of General Surgery – Antalya, Turkey.; 3 University of Health Sciences – Antalya Training and Research Hospital – Department of Pathology – Antalya, Turkey.; 4 University of Health Sciences – Antalya Training and Research Hospital – Department of Medical Biochemistry – Antalya, Turkey.

**Keywords:** Liver, Cholestasis, Ischemia, Perfusion

## Abstract

**Purpose:**

To evaluate the potential protective effects of naringin on liver by oxidative parameters and signal peptide-CUB-EGF-like domain-containing protein (SCUBE)-1 and 2 in an experimental cholestatic liver ischemia reperfusion (IR) model.

**Methods:**

Twenty-four female rats were divided into three groups; sham, control, and treatment. Groups 2 and 3 underwent bile duct ligation, and one week later, 45 min of ischemia and 1 hour of relaparotomy and reperfusion were performed. To the treatment group, naringin was administered through relaparotomy. Liver tissue and blood samples were taken. Alanine aminotransferase (ALT), aspartate aminotransferase (AST), alkaline phosphatase (ALP), total bilirubin (TBIL), direct bilirubin (DBIL), ischemia-modified albumin (IMA), SCUBE-1, SCUBE-2, total antioxidant status (TAS), and total oxidant status (TOS) levels were also examined.

**Results:**

Serum ALT, AST, ALP, TBIL, DBIL, and IMA levels were higher in groups 2 and 3. There was no significant difference in terms of SCUBE-1 and 2 levels (*p* > 0.05). TAS was the highest in group 3, and TOS was the highest in group 2 and lower in group 3. In histopathological analysis, all parameters were statistically significant between group 3 and the other groups (*p* < 0.05).

**Conclusion:**

Naringin has promising results in the treatment of experimental IR injury in cholestatic liver due to its antioxidant effects. We think that it can be used in clinical studies after more comprehensive studies investigating its effects on IR damage in cholestatic liver.

## Introduction

Ischemia is defined as the interruption or reduction of blood flow to the tissue for any reason. Reperfusion refers to the normalization of blood flow in ischemic tissue. Ischemia-reperfusion (IR) injury is the damage caused by free oxygen radicals (FOR) generated during reperfusion, which occurs in an attempt to restore tissue viability^1^.

Clinical conditions such as trauma, shock, liver surgery, and transplantation are among the most common causes of liver IR injury^2^. Several mechanisms have been identified regarding IR injury. Excessive FOR are produced due to disruption of the cytoskeleton and membrane phospholipids, resulting from changes in mitochondrial oxidative phosphorylation, decreased adenosine triphosphate (ATP), increased intracellular calcium, and activation of proteases and phosphatases, all of which contribute to oxidative stress^3^^,^ 4. The status of oxidants can be determined by measuring the total oxidant status (TOS)^5^^,^ 6, while the total antioxidant status (TAS) reflects the organism’s total defense against FOR in plasma. Measuring TAS and TOS can provide useful insights into oxidative status^7^.

In the presence of cholestasis, the liver becomes more susceptible to reperfusion injury^8^^,^ 9. When bile flow is interrupted, sinusoidal endothelial cell damage accelerates, neutrophil accumulation increases, and Kupffer cell activation is stimulated9. Activated neutrophils and Kupffer cells are the primary sources of FOR during oxidative stress^10^^,^ 11. Cholestasis is commonly observed in surgeries for hepatopancreatobiliary diseases, making cholestatic IR models particularly relevant for studying these conditions.

Naringin (4’5,7-trihydroxyflavanone 7-rhamnoglucoside) is a bioflavonoid, polyphenolic compound found in various fruits, including grapefruit, and citrus fruits. After oral administation, it hydrolyzed by the intestinal microflora to form naringenin, a potent and absorbable major metabolite^12^. Naringenin has a range of pharmacologic properties, including antioxidant, anti-inflammatory, anti-ulcer, anti-diabetic, and neuroprotective effects^13^^–^ 15. Recent experimental studies have shown that naringin possesses antioxidant properties and can prevent oxidative stress caused by IR or H_2_O_2_ in various tissues^16^^,^ 17.

The aim of this experimental study was to investigate the effects of naringin on experimental IR injury in cholestatic liver, through the evaluation of oxidative stress and histopathological changes. To our knowledge, this is the first study in the literature to examine the effect of naringin on IR-induced cholestatic liver injury.

## Methods

Approval for this study was granted by Antalya Akdeniz University Animal Research Ethics Committee (2024.05.005). All procedures were conducted in accordance with the principles of the National Guide for the Experimental Use of Laboratory Animals at Akdeniz University Experimental Medicine and Animal Laboratory.

### Animals

In this study, 24 female Wistar albino rats, weighing between 200–250 grams, were used. The rats were housed in cages wherewith controlled environmental conditions (temperature = 23°C and humidity = 55.5%) during both the preoperative and postoperative periods. They were fed with standard laboratory chow and provided with tap water. Access to food was withheld for 12 hours prior to anesthesia, and access to water was withheld for 2 hours prior to anesthesia.

### Surgery and experimental protocol

This study aimed to investigate the effects of naringin on oxidative parameters and signal peptide-CUB-EGF-like domain-containing protein (SCUBE)-1 and 2 in IR damage in cholestatic liver. The rats were randomly divided into three groups:

- Group 1 (control): only laparotomy was performed on the rats in this group, with no additional treatments applied. At the end of the study, the rats were sacrificed after being kept for 1 hour and 45 min. Blood and tissue samples were then collected for analysis;
- Group 2: after laparotomy, the common bile duct was dissected from the surrounding tissue and tied with 3/0 silk suture. Following this, each abdominal incision was closed with a 3/0 polyproline suture. The rats were allowed to feed. On the 7th postoperative day, a relaparotomy was performed, and a vascular clamp was placed on the portal vein and hepatic artery for 45 min to induce ischemia. After the clamp was released, hepatic reperfusion was carried out for 60 min. No other interventions or medications were administered. Liver tissue and blood samples from the aorta were collected for histopathological and biochemical analysis, and the rats were then sacrificed;
- Group 3 (IR + naringin): similar to group 2, after laparotomy, the common bile duct was dissected from the surrounding tissues and tied with 3/0 silk suture. The abdominal incision was closed with 3/0 polyproline suture. The rats were allowed to feed. On the seventh postoperative day, 80 mg/kg naringin (Sigma Aldrich, St. Louis, Missouri, United States of America) was administered intraperitoneally. After waiting 10 min, a relaparotomy was performed, and a vascular clamp was placed on the portal vein and hepatic artery for 45 min to induce ischemia. The clamp was then released, and reperfusion was performed for 1 hour. Liver tissue and blood samples from the aorta were collected for histopathological and biochemical analyses, and the rats were subsequently sacrificed.

### Biochemical analysis

#### Routine parameters

Rat serum albumin, alanine aminotransferase (ALT), aspartate aminotransferase (AST), alkaline phosphatase (ALP), total bilirubin (TBIL), and direct bilirubin (DBIL) levels were determined using a commercially available kit on an autoanalyzer (Beckman AU5800; Beckman, Coulter Diagnostics, Brea, CA, United States of America).

#### Measurement of SCUBE-1 and 2

Rat serum SCUBE-1 and SCUBE-2 levels were measured using a commercially available enzyme-linked ımmunosorbent assay (ELISA) kit (Bioassay Technology Laboratory, Shanghai, China; catalog no. E0948Ra) according to the manufacturer’s instructions. The sensitivity of the kit was 0.35 ng/mL for SCUBE-1 and 0.07 ng/mL for SCUBE-2. The inter- and intra-assay coefficients of variation for SCUBE-1 and 2 were both < 10%. Test results were expressed as ng/mL.

#### Measurement of serum total antioxidant status

Serum TAS levels were analyzed using the automatic colorimetric measurement method^18^. In this method, antioxidants in the sample reduce the dark blue-green colored 2,2′-azino-bis(3-ethylbenzthiazoline-6-sulfonic acid) (ABTS) radicals to the colorless, reduced ABTS form. The absorbance change at 660 nm is related to the total antioxidant level in the sample. This method measures the antioxidative effect of the sample against the strong free radical reactions initiated by the hydroxyl radical. The results are expressed as micromolar Trolox equivalents per L (μmol Trolox equivalent/L).

#### Measurement of serum total oxidant status

Serum TOS levels were analyzed using the automatic colorimetric measurement method19. In this method, oxidants in the sample oxidize the ferrous ion-chelated complex to ferric ion, which form a colored complex with chromogenicity in an acidic environment. The color intensity, which can be measured by spectrophotometry, is directly related to the total amount of oxidant molecules present in the sample. The results are expressed in micromolar hydrogen peroxide equivalents per L (μmol H_2_O_2_ equivalent/L).

#### Measurement of ischemia modified albumin

The reduced cobalt-albumin binding capacity (IMA level) was measured using a rapid, colorimetric method^20^. Briefly, 200 μL of serum was transferred to glass tubes, and 50 μL of 0.1% CoCl_2_ * 6H_2_O (lot S38901-248, Sigma Aldrich, St Louis, MO, United States of America) was added. After gentle shaking, the mixture was incubated for 10 min to ensure sufficient cobalt-albumin binding. Then, 50 μL of 1.5 mg/mL dithiothreitol (DTT) (lot D5545-1G, Sigma Aldrich, St Louis, MO, United States of America) was added as the coloring agent. After 2 min, 1 mL of 0.9% NaCl was added to stop the binding between cobalt and albumin. A blank was prepared for each sample: during the DTT addition step, 50 μL of distilled water was used instead of 50 μL of 1.5 mg/mL DTT to obtain a DTT-free blank. Absorbances were recorded at 470 nm with a spectrophotometer (UV1201, Shimadzu, Kyoto, Japan). Color formation in samples with DTT was compared to color formation in blank tubes, and the results were expressed in absorbance units.

### Histopathological evaluation

Liver samples removed from rats were fixed in 10% neutral formalin solution. After 48 hours of fixation, tissues were dehydrated through a series of graded alcohols, embedded in paraffin, and cut into 4-μm sections using a microtome (Leica RM 2125, Leica Microsystems Nussloch GmbH, Germany). The sections were then stained with hematoxylin and eosin (H&E). Additionally, Masson trichrome stain was used to evaluate fibrosis. These preparations were examined blindly by the pathologist under a light microscope.

Liver damage was assessed for severity using an ordinal scale as follows:

- Grade 0: minimal or no evidence of damage;
- Grade 1: mild injury with cytoplasmic vacuolation and focal nuclear pyknosis;
- Grade 2: moderate to severe injury with nuclear pyknosis, cytoplasmic hypereosinophilia, and loss of intercellular boundaries;
- Grade 3: severe necrosis with rupture of hepatic cords, bleeding, and neutrophil infiltration^21^.

### Statistical analysis

Descriptive statistics included frequency, percentage, mean, standard deviation, median, minimum, and maximum values. If the data were not normally distributed, the Kruskal-Wallis H test was used. In the presence of a significant difference, the Dunn-Bonferroni procedure was applied. Since the numerical variables did not follow a normal distribution, the Spearman correlation coefficient was used to test the relationship between them. Statistical Package for the Social Sciences (SPSS) version 23.0 (IBM Corp., Armonk, NY, United States of America) was used to analyze the research data. A value of *p* < 0.05 was considered statistically significant.

## Results

The analysis results of the biochemical parameters of the groups are presented in Tables 1 and 2. Serum levels of ALT, AST, ALP, IMA, TBIL, and DBIL were low in the sham group and high in the IR and IR + naringin groups. There was no statistically significant difference between these two groups (*p* > 0.05). Serum levels of SCUBE-1 and 2 did not differ significantly between the groups.

**Table 1:** Laboratory findings of study groups (the results are presented as median and 95% confidence interval). Kruskal-Wallis’ test was performed, and those found to be significant were evaluated with Dunn’s multiple comparisons test.

Parameter	Sham (n = 7) (a)	IR (n = 7) (b)	IR + naringin (n = 7) (c)	*p*-value
SCUBE-1 (ng/mL)	8.79 (6.46–12.27)	8.25 (3.55–16.34)	9.15 (5.1–17.71)	0.96
SCUBE-2 (ng/mL)	0.29 (0.23–0.36)	0.25 (0.13–0.65)	0.45 (0.12–1.14)	0.31
TAS* (μmol Trolox equiv./L)	2.37(1.94–3.99)	1.58 (1.41–1.88)	4.37 (3.94–5.59)	< 0.01
TOS**(μmol Trolox equiv./L)	1.08 (1.01–1.11)	2.12 (1.08–2.14)	1.18 (1.04–1.63)	0.03
IMA*** (ABSU)	0.35 (0.246–0.45)	0.52 (0.44–0.98)	0.48 (0.32–0.58)	< 0.01
ALT# (U/L)	37 (27–65)	422 (162–876)	172 (97–694)	< 0.01
AST## (U/L)	98 (75–117)	1898 (948–4434)	560 (320–2,766)	< 0.01
ALP### (U/L)	83.5 (65–115)	263 (192–444)	183 (120–545)	< 0.01
TBIL& (mg/dL)	0.17 (0.12–0.19)	11.4 (2.8–17)	11.07 (0.12–14.02)	< 0.01
DBIL&& (mg/dL)	0.025 (0.02–0.06)	6.84 (1.26–9.4)	6.62 (0.04–8.6)	< 0.01

IR: ischemia reperfusion; SCUBE-1: signal peptide-CUB-EGF-like domain-containing protein 1; SCUBE-2: signal peptide-CUB-EGF-like domain-containing protein 2; TAS: total antioxidant status; TOS: total oxidant status; IMA: ischemia-modified albumin; ALT: alanine aminotransferase; AST: aspartate aminotransferase; ALP: alkaline phosphatase; TBIL: total bilirubin; DBIL: direct bilirubin. Multiple comparisons test:

* c > a (*p* = 0.03), c > b (*p* < 0.01)

** b > a (*p* = 0.03);

*** b > a (*p* < 0.01);

# b > a (*p* = 0.04), c > a (*p* < 0.01);

## b > a (*p* < 0.01);

### b > a (*p* = 0.02), c > a (*p* < 0.01);

& b > a (*p* < 0.01), c > a (*p* < 0.01);

&& b > a (*p* = 0.01); c > a (*p* < 0.01).

Source: Elaborated by the authors.

**Table 2:** Laboratory findings of study groups according to the Spearman correlation analysis.

Bivariate correlation	r	*p*-value
SCUBE-2 & IMA	0.409	0.06
SCUBE-2 & ALT	0.415	0.06
SCUBE-2 & AST	0.384	0.07
SCUBE-2 & ALP	0.439	0.04
SCUBE-2 & TBIL	0.414	0.05
TAS & TOS	-0.615	< 0.01
TAS & TBIL	0.797	< 0.01
TAS & DBIL	0.705	< 0.01
IMA & ALT	0.470	0.03
IMA & AST	0.503	0.02
IMA & DBIL	0.560	< 0.01
ALT & AST	0.927	< 0.01
ALT & ALP	0.774	< 0.01
ALT & TBIL	0.719	< 0.01
ALT& DBIL	0.789	< 0.01
AST & ALP	0.770	< 0.01
AST & TBIL	0.749	< 0.01
AST & DBIL	0.826	< 0.01
ALP & TBIL	0.722	< 0.01
ALP & DBIL	0.703	< 0.01
TBIL & DBIL	0.905	< 0.01

r: Spearman rank correlation coefficient; SCUBE-2: signal peptide-CUB-EGF-like domain-containing protein 2; IMA: ischemia-modified albumin; ALT: alanine aminotransferase; AST: aspartate aminotransferase; ALP: alkaline phosphatase; TBIL: total bilirubin; TAS: total antioxidant status; TOS: total oxidant status; DBIL: direct bilirubin. Source: Elaborated by the authors.

When examining the TAS, the antioxidant capacity of the naringin administered group was significantly higher. Regarding TOS, all three groups showed significantly differences from each other (*p* = 0.03). The sham group had the lowest TOS values, while the IR group had the highest TOS values. The TOS values in the naringin group were significantly higher than in the control group, but significantly lower than in the IR group.

**Figure 1:**
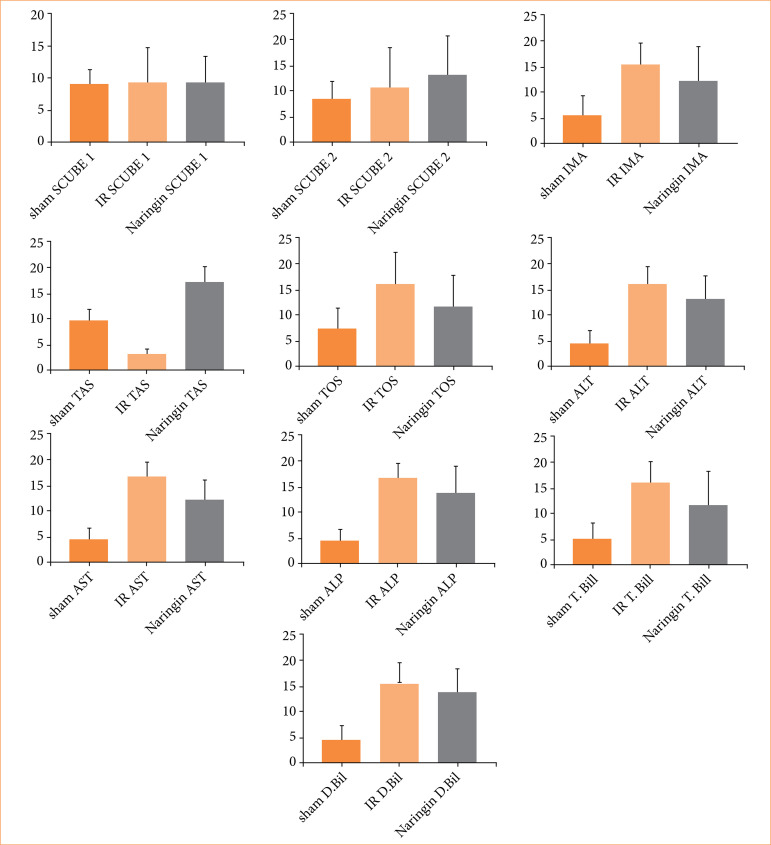
Distribution of biochemical parameters. No significant difference was observed between the groups in terms of SCUBE-1 and 2 levels.

SCUBE-2: signal peptide-CUB-EGF-like domain-containing protein 2; IR: ischemia reperfusion; IMA: ischemia-modified albumin; TAS: total antioxidant status; TOS: total oxidant status; ALT: alanine aminotransferase; AST: aspartate aminotransferase; ALP: alkaline phosphatase; TBIL: total bilirubin; DBIL: direct bilirubin. Source: Elaborated by the authors.

The comparison and distribution of biochemical parameters are shown in Fig. 1. No significant difference was observed between the groups in terms of SCUBE-1 and 2 levels. There was no correlation between SCUBE-2 and any other variables. It was found that TAS had a moderate positive correlation with TOS, TBIL, and DBIL, while IMA showed a weak positive correlation with ALT, AST, and DBIL.

The relationship between the groups in terms of liver damage, cholestasis, and fibrosis scores was investigated by histopathological examination. Subjects were classified based on the degree of liver damage into grade 0, grade 1, grade 2, and grade 3. A statistical relationship was found between liver injury scores and experimental groups. A significant difference was observed between the groups in terms of fibrosis score (*p* = 0.005).

**Figure 2:**
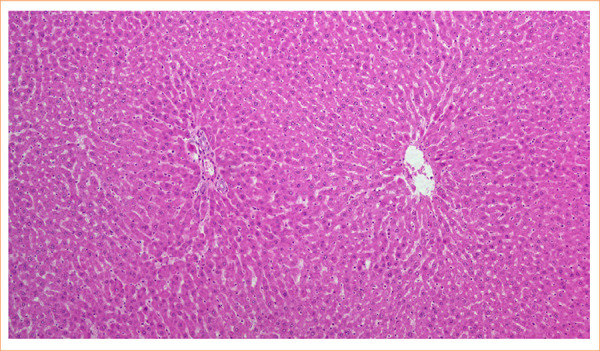
Normal liver parenchyma features were observed in the rats of the control group (grade 0). (X100, hematoxylin and eosin).

Source: Elaborated by the authors.

No cholestasis or fibrosis was observed in the histopathological examination of the liver samples in group 1. Liver damage was minimal or absent in controls, and no parenchymal damage was observed in group 1. Normal liver parenchyma features were observed in the rats of the control group (Fig. 2).

**Figure 3:**
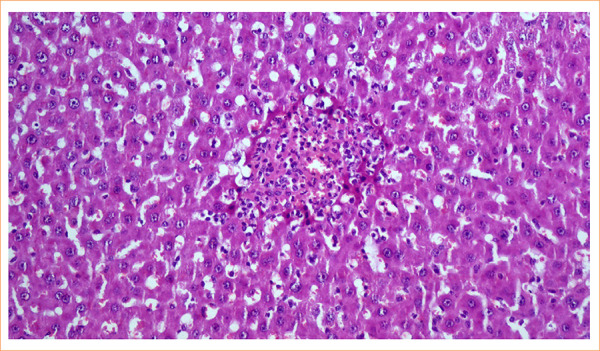
Bleeding and neutrophil exudation were observed at the site of hepatocyte cords that suffered necrosis due to ischemic injury in the ischemia reperfusion group (grade 3) (X200, hematoxylin and eosin).

Source: Elaborated by the authors.

In the histopathological examination of the cases in group 2, bile duct proliferation, mild to moderate neutrophilic infiltration, and dilatation due to fibrosis were detected. In the IR group, grade 3 injury was observed, with bleeding and neutrophil exudation at the site of hepatocyte cords that had undergone necrosis due to ischemic damage (Fig. 3).

**Figure 4:**
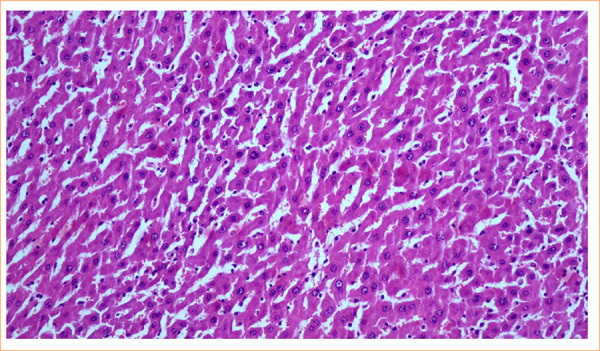
Dissociation of the connections between hepatocytes, hypereosinophilia in the cytoplasm of hepatocytes, nuclear pyknosis, and mitotic figures were observed in the naringin treatment group (grade 2). (X200, hematoxylin and eosin).

Source: Elaborated by the authors.

Histopathological examination revealed less neutrophil infiltration and bile duct proliferation in the portal areas of group 3. Grade 2 dissociation damage in the connections between hepatocytes, hypereosinophilia, nuclear pyknosis, and mitotic figures were observed in the cytoplasm of hepatocytes of naringin treatment group (Fig. 4).

## Discussion

IR injury to the liver is common in complex surgical procedures, major trauma, and transplantation cases. While efforts have been made to reduce this damage, preventing its development remains challenging. It is emphasized that antioxidant substances should be administered before ischemia occurs. As no effective method has been found yet, experimental studies are ongoing.

The liver has significant blood flow to maintain many synthetic and secretory functions making it highly sensitive to IR. Cellular damage is observed 2–6 hours after reperfusion in the early period, while neutrophil dominance occurs in the later stages. In the early phase, free oxygen radical-mediated damage is prominent, whereas in the later phase, neutrophil-mediated inflammatory response predominates^22^. Another key mechanism in IR injury is the disruption of intracellular Ca^+2^, Na^+^, H^+^ balance due to decreased mitochondrial ATP production. Increased intracellular Ca^2+^ promotes the production of FOR and triggers apoptotic mechanisms. Cholestasis, a clinical condition resulting from the cessation or slowing of bile flow due to obstruction of the intra- or extrahepatic bile ducts, can also lead to FOR release by increasing Kupffer cell activation. When IR injury occurs in the presence of cholestasis, the damage is significantly more severe^23^. Many surgeons expect preoperative bilirubin levels to decrease in patients with very high bilirubin levels prior to elective surgeries.

There are many studies in the literature on liver IR damage and its prevention. However, the liver IR injury model in cholestatic rats is a relatively newer experimental model, and thus, there are fewer studies available^23^^–^ 25.

Naringin has been reported to exhibit antioxidant, superoxide scavenging, anti-inflammatory, anti-apoptotic, anti-diabetic, and hepato-protective effects^26^^–^ 31.

We investigated the potential protective effect of the antioxidant naringin on the liver by analyzing oxidative parameters and SCUBE-1 and 2. To our knowledge, this is the first study to explore the hepatoprotective effects of naringin and its impact on the recently described glycoproteins SCUBE-1 and 2 in cholestatic rats with obstructive jaundice and IR injury. The most significant finding of our study is that naringin has a hepatoprotective effect in rats with experimentally induced IR damage in the cholestatic liver. Both histopathological and biochemical results showed better outcomes in the naringin-treated group compared to the control group in terms of liver function and tissue health.

SCUBE-1 is a novel biomarker. Lindemann and Gawaz^32^ identified the SCUBE-1 cDNA fragment localized to chromosome 22q13 in the endothelium and demonstrated a fibrin-rich region within the organized thrombus in platelets. SCUBE-1 accumulates in alpha granules of platelets and is activated on the cell surface upon platelet stimulation. It has been reported that SCUBE-1 levels increase 6 hours after platelet activation, and plasma levels can be measured for three or four days. SCUBE-1 levels have been found to rise in cases of pulmonary thromboembolism and experimentally induced ischemic stroke. Similarly, SCUBE-1 levels have been reported to increase in 2 hours after acute mesenteric ischemia and as a result of platelet dysfunction in hemodialysis patients^33^^–^ 36.

In this study, SCUBE-1 and 2 levels were not elevated in groups 2 and 3, where cholestasis was induced. The levels were similar between groups and did not show statistically significance (*p* = 0.96, *p* = 0.31).

SCUBE-2 is a vascular endothelial cell surface protein closely related to SCUBE-1 and has been shown to play a role in the development and progression of coronary artery atherosclerosis^37^. One study reported a lower recurrence rate and better survival in patients with SCUBE-2 positive colorectal cancer^38^. The relationship between SCUBE-1, 2 and 3 levels and clinical findings was investigated in patients with Hashimoto’s thyroiditis, psoriasis, and systemic sclerosis caused by angiogenesis and inflammatory reactions^38^^,^ 39. It has been demonstrated that SCUBE-1 levels are higher in patients with hypothyroidism due to Hashimoto’s thyroiditis compared to healthy individuals^40^. Additionally, SCUBE-2 has been suggested to play a role in endothelial dysfunction and reactive oxygen species (ROS) accumulation in conditions such as diabetes, hyperglycemia, and dyslipidemia, all of which are associated with increased vascular complications^41^.

When we look at the TAS, we see that the naringin-administered group is significantly higher than the group 1 and IR groups. When we examine the TOS, we observe that all three groups differ significantly from each other. Group 1 has the lowest oxidant capacity, while the IR group has the highest. The naringin group was statistically significantly lower than the IR group. Based on these data, we could conclude that naringin increases antioxidant capacity and reduces oxidative stress.

IMA is an albumin that undergoes structural changes as a result of ischemia. In addition to ischemia, IMA levels may increase in conditions such as acute coronary syndrome, cerebrovascular diseases, liver failure, end-stage renal disease, severe trauma, pulmonary embolism, sepsis, diabetes, systemic sclerosis, some neoplastic diseases, and infections^42^^–^ 44. In a study investigating IMA in chronic liver disease, 43 individuals with chronic liver disease were compared to 28 healthy controls. The results showed that IMA values were significantly higher in the chronic liver disease group, but AST and ALT levels were not correlated with this increase. IMA was inversely related to albumin levels^45^.

In another study involving 33 healthy children and 33 children with chronic liver disease, a correlation was found between fibrous scoring and IMA, but no relationship was observed with other biochemical parameters^46^. In an experimental study on acute mesenteric ischemia, an increase in IMA levels was observed at 0, 1, 3 and 6 hours, but no significant difference was found between these time points^47^. In our study, the IMA values were low in the first group, while the IMA values in the IR group and the naringin group were higher. There was a statistically significant difference between the groups.

Although there are various methods to assess parenchymal damage in liver IR injury, the most commonly used measurements are serum AST, ALT, and lactate dehydrogenase (LDH) values^48^. ALT is a cytosolic enzyme primarily found in the liver, whereas AST is located in the mitochondria and targets present in the liver, skeletal muscles, and kidneys. ALP activity increases due to obstruction or inflammation of the biliary tract. Its increase in the blood is largely attributed to infiltration of ALT and AST into the hepatocytes, which is considered a marker of liver damage or dysfunction. In this study, naringin was observed to have a protective effect on 5-FU-induced liver and kidney damage^49^. In our study, we observed that the values in the first group and in naringin one were low, while the values in the IR group were high. A statistically significant difference was found when comparing the IR group to the first group and naringin one. The administration of naringin resulted in significant recovery and a notable reduction in the activity of these enzymes, suggestive of hepatotoxicity in animals exposed to IR injury.

In the case of cholestasis, serum DBIL levels and ALP levels are the most investigated parameters. A recent study found that oral administration of lupeol, naringin, and their combination demonstrated curative potential against bile duct ligation induced cardiac injury in rats. This effect was achieved by reducing oxidative stress and inflammatory reactions, which led to decreased heart necrosis/myocardial infarction and increased myocardial activity^50^. When the groups were compared, TBIL and DBIL levels and ALP were lower in group 1 and higher in both the IR and N groups. Again, no statistically significant difference was observed between the IR and N groups.

## Conclusion

This study is the first one to examine the effect of naringin on IR injury in a cholestatic liver model. Naringin treatment significantly reduced liver damage, inflammation, fibrosis, and necrosis, as well as improved antioxidant capacity. Biochemical analysis revealed lower levels of TOS, liver enzymes, and bilirubin in the naringin-treated group. Histopathological findings showed less cellular damage, such as neutrophilic infiltration, necrosis, and bile duct proliferation in the naringin group compared to the cholestatic group. These results suggested that naringin has a hepatoprotective effect against IR injury in cholestatic liver. Further research is needed to better understand its mechanisms and potential clinical applications.

## Data Availability

All data sets were generated or analyzed in the current study.

## References

[1] Lu TF, Yang TH, Zhong CP, Shen C, Lin WW, Gu GX, Xia Q, Xu N (2018). Dual effect of hepatic macrophages on liver ischemia and reperfusion injury during liver transplantation. Immune Netw.

[2] Nastos C, Kalimeris K, Papoutsidakis N, Tasoulis MK, Lykoudis PM, Theodoraki K, Nastou D, Smyrniotis V, Arkadopoulos N (2014). Global consequences of liver ischemia/reperfusion injury. Oxid Med Cell Longev.

[3] Aragno M, Cutrin JC, Mastrocola R, Perrelli MG, Restivo F, Poli G, Danni O, Boccuzzi G (2003). Oxidative stress and kidney dysfunction due to ischemia/reperfusion in rat: attenuation by dehydroepiandrosterone. Kidney Int.

[4] Jaeschke H, Woolbright BL (2012). Current strategies to minimize hepatic ischemia-reperfusion injury by targeting reactive oxygen species. Transplant Rev.

[5] Kohen R, Nyska A (2002). Oxidation of biological systems: oxidative stress phenomena, antioxidants, redox reactions, and methods for their quantification. Toxicol Pathol.

[6] Czerska M, Mikołajewska K, Zieliński M, Gromadzińska J, Wąsowicz W (2015). Today’s oxidative stress markers. Med Pr.

[7] Aslan M, Cosar N, Celik H, Aksoy N, Dulger AC, Begenik H, Soyoral YU, Kucukoglu ME, Selek S (2011). Evaluation of oxidative status in patients with hyperthyroidism. Endocrine.

[8] Kloek JJ, Marsman HA, van Vliet AK, Gouma DJ, van Gulik TM (2008). Biliary drainage attenuates postischemic reperfusion injury in the cholestatic rat liver. Surgery.

[9] Yoshidome H, Miyazaki M, Shimizu H, Ito H, Nakagawa K, Ambiru S, Nakajima N, Edwards MJ, Lentsch AB (2000). Obstructive jaundice impairs hepatic sinusoidal endothelial cell function and renders liver susceptible to hepatic ischemia/reperfusion. J Hepatol.

[10] Jaeschke H, Farhood A, Smith CW (1990). Neutrophils contribute to ischemia/reperfusion injury in rat liver in vivo. FASEB J.

[11] Datta G, Fuller BJ, Davidson BR (2013). Molecular mechanisms of liver ischemia reperfusion injury: insights from transgenic knockout models. World J Gastroenterol.

[12] Ameer B, Weintraub RA, Johnson JV, Yost RA, Rouseff RL (1996). Flavanone absorption after naringin, hesperidin, and citrus administration. Clin Pharmacol Ther.

[13] Dhalla NS, Elmoselhi AB, Hata T, Makino N (2000). Status of myocardial antioxidants in ischemia-reperfusion injury. Cardiovasc Res.

[14] Sharma AK, Bharti S, Ojha S, Bhatia J, Kumar N, Ray R, Kumari S, Arya DS (2011). Up-regulation of PPARγ, heat shock protein-27 and -72 by naringin attenuates insulin resistance, β-cell dysfunction, hepatic steatosis and kidney damage in a rat model of type 2 diabetes. Br J Nutr.

[15] Singh D, Chopra K (2004). The effect of naringin, a bioflavonoid on ischemia-reperfusion induced renal injury in rats. Pharmacol Res.

[16] Akondi BR, Challa SR, Akula A (2011). Protective effects of rutin and naringin in testicular ischemia-reperfusion induced oxidative stress in rats. J Reprod Infertil.

[17] Kanno S, Shouji A, Asou K, Ishikawa M (2003). Effects of naringin on hydrogen peroxide-induced cytotoxicity and apoptosis in P388 cells. J Pharmacol Sci.

[18] Erel O (2004). A novel automated direct measurement method for total antioxidant capacity using a new generation, more stable ABTS radical cation. Clin Biochem.

[19] Erel O (2005). A new automated colorimetric method for measuring total oxidant status. Clin Biochem.

[20] Bar-Or D, Lau E, Winkler JV (2000). A novel assay for cobalt-albumin binding and its potential as a marker for myocardial ischemia-a preliminary report. J Emerg Med.

[21] Onder A, Kapan M, Gümüş M, Yüksel H, Böyük A, Alp H, Başarili MK, Firat U (2012). The protective effects of curcumin on intestine and remote organs against mesenteric ischemia/reperfusion injury. Turk J Gastroenterol.

[22] Tapuria N, Junnarkar S, Abu-Amara M, Fuller B, Seifalian AM, Davidson BR (2012). Modulation of microcirculatory changes in the late phase of hepatic ischaemia-reperfusion injury by remote ischaemic preconditioning. HPB.

[23] Çakır T, Aslaner A, Tekeli SÖ, Güneş K, Kinaci E, Doğan U, Tekeli F, Akyüz C, Koç S, Yılmaz N (2016). Grape seed protects cholestatic rats liver from ischemia/reperfusion injury. Acta Cir Bras.

[24] Güler S, Aslaner A, Ellidağ HY, Yıldırım Ş, Çakır T (2021). The protective effect of boric acid on cholestatic rat liver ischemia reperfusion injury. Turk J Med Sci.

[25] Dogan U, Yilmaz AH, Yildirim S, Ellidag HY, Aslaner A, Cakir RC, Koctekin B, Karakas BR, Cakir T (2023). The effect of alpha-lipoic acid on oxidative parameters, SCUBE-1 and SCUBE-2 in hepatic ischemia-reperfusion injury in cholestatic rats. Eur Rev Med Pharmacol Sci.

[26] Gopinath K, Sudhandiran G (2012). Naringin modulates oxidative stress and inflammation in 3-nitropropionic acid-induced neurodegeneration through the activation of nuclear factor-erythroid 2-related factor-2 signalling pathway. Neuroscience.

[27] Kanno S, Shouji A, Asou K, Ishikawa M (2003). Effects of naringin on hydrogen peroxide-induced cytotoxicity and apoptosis in P388 cells. J Pharmacol Sci.

[28] Liu Y, Su WW, Wang S, Li PB (2012). Naringin inhibits chemokine production in an LPS-induced RAW 264.7 macrophage cell line. Mol Med Rep.

[29] Prakash A, Shur B, Kumar A (2013). Naringin protects memory impairment and mitochondrial oxidative damage against aluminum-induced neurotoxicity in rats. Int J Neurosci.

[30] Sharma AK, Bharti S, Ojha S, Bhatia J, Kumar N, Ray R, Kumari S, Arya DS (2011). Up-regulation of PPARγ, heat shock protein-27 and -72 by naringin attenuates insulin resistance, β-cell dysfunction, hepatic steatosis and kidney damage in a rat model of type 2 diabetes. Br J Nutr.

[31] Singh D, Chopra K (2004). The effect of naringin, a bioflavonoid on ischemia-reperfusion induced renal injury in rats. Pharmacol Res.

[32] Lindemann S, Gawaz M (2006). SCUBE1--a new scoop in vascular biology?. Cardiovasc Res.

[33] Dirican N, Duman A, Sağlam G, Arslan A, Ozturk O, Atalay S, Bircan A, Akkaya A, Cakir M (2016). The diagnostic significance of signal peptide-complement C1r/C1s, Uegf, and Bmp1-epidermal growth factor domain-containing protein-1 levels in pulmonary embolism. Ann Thorac Med.

[34] Turkmen S, Eryigit U, Karaca Y, Mentese A, Sumer UA, Yulug E, Aksut N, Gazioglu S, Gunduz A (2015). Diagnostic value of plasma signal peptide-Cub-Egf domain-containing protein-1 (SCUBE-1) in an experimental model of acute ischemic stroke. Am J Emerg Med.

[35] Bronze L (2018). SCUBE 1: A novel biomarker related to platelet activation and atherothrombosis. Rev Port Cardiol.

[36] Ulusoy S, Ozkan G, Menteşe A, Yavuz A, Karahan SC, Sümer AU (2012). Signal peptide-CUB-EGF domain-containing protein 1 (SCUBE1) level in hemodialysis patients and parameters affecting that level. Clin Biochem.

[37] Ali H, Emoto N, Yagi K, Vignon-Zellweger N, Nakayama K, Hatakeyama K, Asada Y, Rikitake Y, Hirata K (2013). Localization and characterization of a novel secreted protein, SCUBE2, in the development and progression of atherosclerosis. Kobe J Med Sci.

[38] Song Q, Li C, Feng X, Yu A, Tang H, Peng Z, Wang X (2015). Decreased expression of SCUBE2 is associated with progression and prognosis in colorectal cancer. Oncol Rep.

[39] Gündüz İ, Batmaz İ, Bozan T, Ekinci A, Cevik R (2020). The relationship of serum SCUBE-1, -2 and -3 levels with clinical findings and ultrasonographic skin thickness in systemic sclerosis patients. Int J Rheum Dis.

[40] Bilir B, Soysal-Atile N, Ekiz Bilir B, Yilmaz I, Bali I, Altintas N, Baykiz D, Aydin M, Guldiken S (2016). Evaluation of SCUBE-1 and sCD40L biomarkers in patients with hypothyroidism due to Hashimoto’s thyroiditis: a single-blind, controlled clinical study. Eur Rev Med Pharmacol Sci.

[41] Ali H (2020). SCUBE2, vascular endothelium, and vascular complications: A systematic review. Biomed Pharmacother.

[42] Yin M, Liu X, Chen X, Li C, Qin W, Han H, Guo H, Yang H, Cao D, Du Z, Wu D, Wang H (2017). Ischemia-modified albumin is a predictor of short-term mortality in patients with severe sepsis. J Crit Care.

[43] Gaze DC (2009). Ischemia modified albumin: a novel biomarker for the detection of cardiac ischemia. Drug Metab Pharmacokinet.

[44] Sbarouni E, Georgiadou P, Voudris V (2011). Ischemia modified albumin changes - review and clinical implications. Clin Chem Lab Med.

[45] Kumar PA, Subramanian K (2016). The role of ischemia modified albumin as a biomarker in patients with chronic liver disease. J Clin Diagn Res.

[46] Cakir M, Karahan SC, Mentese A, Sag E, Cobanoglu U, Polat TB, Erduran E (2012). Ischemia-modified albumin levels in children with chronic liver disease. Gut Liver.

[47] Dundar ZD, Cander B, Gul M, Karabulut KU, Girisgin S (2010). Serum ischemia-modified albumin levels in an experimental acute mesenteric ischemia model. Acad Emerg Med.

[48] Aslaner A, Çakır T, Çelik B, Doğan U, Akyüz C, Baştürk A, Polat C, Gündüz U, Mayir B, Şehirli AÖ (2015). The protective effect of intraperitoneal medical ozone preconditioning and treatment on hepatotoxicity induced by methotrexate. Int J Clin Exp Med.

[49] Gelen V, Şengül E, Yıldırım S, Atila G (2018). The protective effects of naringin against 5-fluorouracil-induced hepatotoxicity and nephrotoxicity in rats. Iran J Basic Med Sci.

[50] Alam F, Kharya AK, Srivastav RK, Akhtar J, Khan MI, Ahmad M (2023). Synergetic effect of lupeol and naringin against bile duct ligation induced cardiac injury in rats via modulating nitrite level (eNos) and NF-kB/p65 expression. Drug Res.

